# Fast Li-Ion Conduction in Spinel-Structured Solids

**DOI:** 10.3390/molecules26092625

**Published:** 2021-04-30

**Authors:** Jan L. Allen, Bria A. Crear, Rishav Choudhury, Michael J. Wang, Dat T. Tran, Lin Ma, Philip M. Piccoli, Jeff Sakamoto, Jeff Wolfenstine

**Affiliations:** 1 Energy Sciences Division, Sensors & Electron Devices Directorate, US Army Research Laboratory, Adelphi, MD 20783, USA; dat.t.tran4.civ@mail.mil (D.T.T.); liam.l.ma.civ@outlook.com (L.M.); 2 Department of Chemistry, Howard University, Washington, DC 20059, USA; bria.a.crear@gmail.com; 3 Department of Materials Science and Engineering, University of Michigan, Ann Arbor, MI 48109, USA; rishavc@umich.edu (R.C.); micwan@umich.edu (M.J.W.); jeffsaka@umich.edu (J.S.); 4 Department of Geology, University of Maryland, College Park, MD 20742, USA; piccoli@umd.edu; 5 Solid Ionic Consulting, 9223 Matthews Ave, Seattle, WA 98115, USA; jeffyspeak@outlook.com

**Keywords:** solid electrolyte, fast Li^+^ ion conductor, Li-ion battery, spinel, solid-state battery, cathode-electrolyte interface

## Abstract

Spinel-structured solids were studied to understand if fast Li^+^ ion conduction can be achieved with Li occupying multiple crystallographic sites of the structure to form a “Li-stuffed” spinel, and if the concept is applicable to prepare a high mixed electronic-ionic conductive, electrochemically active solid solution of the Li^+^ stuffed spinel with spinel-structured Li-ion battery electrodes. This could enable a single-phase fully solid electrode eliminating multi-phase interface incompatibility and impedance commonly observed in multi-phase solid electrolyte–cathode composites. Materials of composition Li_1.25_M(III)_0.25_TiO_4_, M(III) = Cr or Al were prepared through solid-state methods. The room-temperature bulk Li^+^-ion conductivity is 1.63 × 10^−4^ S cm^−1^ for the composition Li_1.25_Cr_0.25_Ti_1.5_O_4_. Addition of Li_3_BO_3_ (LBO) increases ionic and electronic conductivity reaching a bulk Li^+^ ion conductivity averaging 6.8 × 10^−4^ S cm^−1^, a total Li-ion conductivity averaging 4.2 × 10^−4^ S cm^−1^, and electronic conductivity averaging 3.8 × 10^−4^ S cm^−1^ for the composition Li_1.25_Cr_0.25_Ti_1.5_O_4_ with 1 wt. % LBO. An electrochemically active solid solution of Li_1.25_Cr_0.25_Mn_1.5_O_4_ and LiNi_0.5_Mn_1.5_O_4_ was prepared. This work proves that Li-stuffed spinels can achieve fast Li-ion conduction and that the concept is potentially useful to enable a single-phase fully solid electrode without interphase impedance.

## 1. Introduction

Interest in solid-state electrolytes has intensified owing to the discovery of fast Li-ion conduction in the cubic garnet structure [1], the continued push for higher energy density batteries and the allure of the safety of an inorganic all solid-state battery. The spinel is a suitable cubic structure to search for fast Li-ion conduction owing to its network of empty edge-shared MO_6_ octahedra bridged by face-shared LiO_4_ tetrahedra, which connect in three dimensions thereby providing a path for 3D Li^+^-ion conduction [2]. In fact, LiMn_2_O_4_ spinel’s favorable mixed electronic-ionic conductivity has enabled its use as a positive electrode [2]. LiMn_2_O_4_ ideally crystallizes in the normal spinel structure in the *Fd*$\bar{3}$*m* space group: Mn occupies the 16d octahedral site, O occupies the 32e position and Li occupies the 8a tetrahedral site which share faces with an empty 16c octahedral site within the spinel’s pseudo-cubic closed packed oxygen framework, thus forming a three-dimensional 8a → 16c → 8a Li ion conduction pathway. Furthermore, the spinel’s cubic unit cell is desirable for solid-state battery application since differences in thermal expansion coefficients of different crystallographic directions in large-grained non-cubic ceramic materials, may lead to micro-cracking [3,4,5] during cooling after densification which is unfavorable for mechanical properties and ionic conductivity [6,7,8,9]. Additionally, the use of a spinel-structured solid electrolyte as a separator in an all solid-state battery and pursuit of high conductivity in the spinel structure can lead to insights that may improve rate capability of spinel structured electrodes for use with liquid based electrolytes or as a catholyte or anolyte in a fully solid-state configuration.

Limited work has been done on oxide spinel structured solid electrolytes. Kawai et al. discovered a conductivity of about 10^−7^ S cm^−1^ for the ordered (P_4_32 space group) spinel LiNi_0.5_Ge_1.5_O_4_ at 63 °C [10]. The Ni and Ge are ordered on the octahedral sites of this compound. In 1985, Thackeray and Goodenough proposed the all-solid all-spinel battery as a means to reduce interfacial impedance at the interface of the solid-state cathode, electrolyte and anode but did not identify a suitable solid-state electrolyte [11]. Rosciano et al. suggested the Li doped MgAl_2_O_4_ spinel as a potential solid-state electrolyte based on high Li diffusivity as measured by nuclear magnetic resonance (NMR) as a means to enable a full spinel concept [12]. However, Djenadic et al. reported that the Li motion is localized in Li doped MgAl_2_O_4_ and therefore the long-range Li conductivity is insufficient for realization of an all-solid all-spinel battery [13].

Spinel is similar to garnet in that in both structures, the occupied tetrahedral and empty octahedral sites form an interconnected 3-D array for Li^+^-ion transport. Conventional garnets are described by the formula A_3_B_3_C_2_O_12_ where A, B, and C have 8, 4, and 6 oxygen coordination, respectively. However, in garnet structured Li_3_Nd_3_Te_2_O_12_, where Li only occupies tetrahedral sites, the Li-ion is practically immobile at room temperature and ionic conductivity can only be measured at elevated temperature, only achieving 10^−5^ S cm^−1^ at 600 °C [14]. In contrast, when additional Li is added to occupy both tetrahedral and octahedral sites in a “Li-stuffed” garnet such as Li_5_La_3_Ta_2_O_12_ [15] room temperature conductivity rises to 1.2 × 10^−6^ S cm^−1^. Further Li leads to even higher conductivity in Li_7−3x_La_3_Zr_2_Al_x_O_12_ which has room temperature ionic conductivity greater than 10^−4^ S cm^−1^ [1]. In this work, we studied a substitutional strategy to form a “Li-stuffed” spinel, with Li occupying both the tetrahedral 8a and octahedral 16d sites, in a somewhat analogous fashion to Li-stuffed cubic garnet. Since we have different energy (tetrahedral and octahedral) sites, the presence of Li on the octahedral sites tends to reduce the energy potential between the two sites making Li motion easier [16]. A room-temperature Li^+^ ionic conductivity greater than 10^−4^ S cm^−1^ is observed for the Li-stuffed spinel. In the composition of highest conductivity, Li_1.25_Cr_0.25_Ti_1.5_O_4_, the 8a tetrahedral site is fully occupied by Li and the 16d octahedral site is 75% occupied by Ti and the remaining 25% split evenly by Li and Cr. The remaining tetrahedral, 8b, and octahedral, 16c, are unoccupied. Li_1.25_Cr_0.25_Ti_1.5_O_4_ contains Ti(IV), which is unstable to Li reduction; however, the use of an interfacial layer such as Li_3_N or a conductive polymer can fix this problem [17] to enable its use with Li or LiC_6_ anodes or more likely the concept can be used in solid solution formation with spinel-structured cathodes, where mixed electronic ionic conductivity is desirable. The integration of solid electrolytes with lithium has progressed rapidly since the report of high conductivity in garnet; however, there has been a lack of progress with cathode–solid electrolyte integration to achieve high ionic and electronic transport through the cathode [18,19]. Unlike conventional Li-ion cells where liquid electrolyte fills the cathode pores providing ion transport and carbon black provides electron transport, cathodes for all solid-state batteries require both ion-conducting as well as electron-conducting additives to enable mixed ionic/electronic transport. However, these solid–solid interfaces have high resistance compared to solid–liquid interfaces, and transport through composite cathodes is a major challenge to enable all solid-state batteries [20]. In a first small step towards application of the concept as solid-state catholyte, we show a solid solution of a Li-stuffed, spinel-structured electrolyte and the LiNi_0.5_Mn_1.5_O_4_ high voltage, spinel-structured positive electrode material to be an electrochemically active cathode material in a liquid-containing cell thus showing that no high resistance solid–solid interfaces are formed.

## 2. Results

### 2.1. X-ray Diffraction (XRD) and Structural Refinement

**Figure 1:**
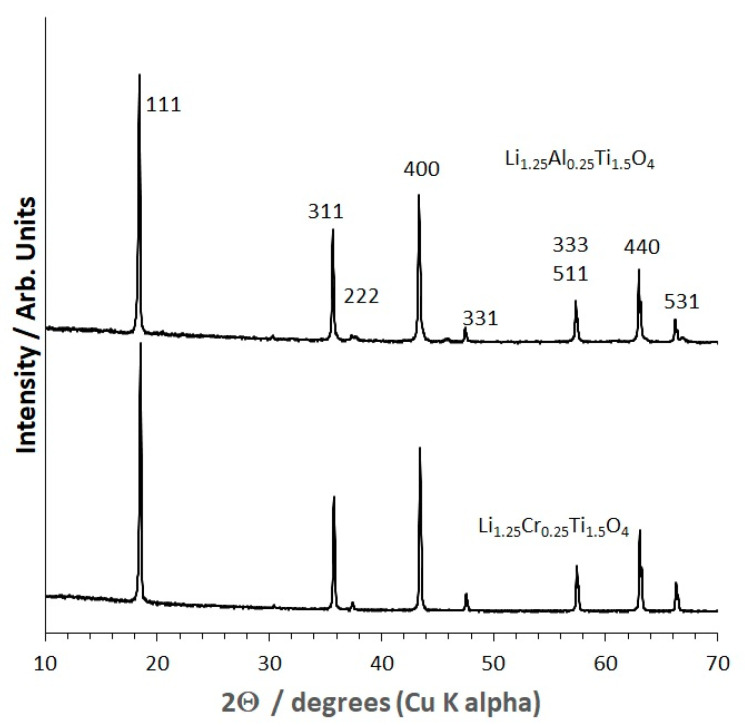
XRD of Li_1.25_Cr_0.25_Ti_1.5_O_4_ solid electrolyte powder (**bottom**) and Li_1.25_Al_0.25_Ti_1.5_O_4_ solid electrolyte powder (**top**). XRD peaks are indexed to the *Fd*$\bar{3}$*m* spinel structure.

**Figure 2:**
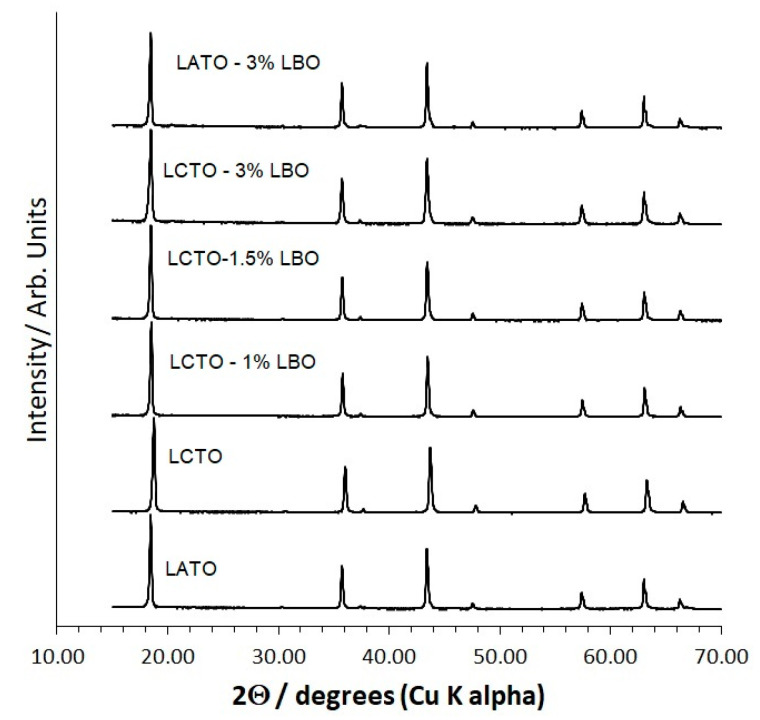
XRD of hot-pressed pellets of Li_1.25_Cr_0.25_Ti_1.5_O_4_ (LCTO) and Li_1.25_Al_0.25_Ti_1.5_O_4_ (LATO) with variable amounts of Li_3_BO_3_ (LBO). Pellets were ground to a powder prior to XRD data collection.

The XRD patterns of the Li_1.25_Cr_0.25_Ti_1.5_O_4_ (LCTO, bottom) and Li_1.25_AlTi_1.5_O_4_ (LATO, top) powders are shown in Figure 1. The patterns are indexed to the cubic spinel structure, space group, *Fd*$\bar{3}$*m*. The LATO pattern has two small, unidentified peaks at 39.64 and 46.18° 2Θ which disappear after hot-pressing (Figure 2). The lattice parameters determined from Rietveld refinement and using Si as internal peak position standard are 8.3440 Å and 8.3574 Å for Li_1.25_Cr_0.25_Ti_1.5_O_4_ (LCTO) and, Li_1.25_AlTi_1.5_O_4_ (LATO), respectively. Since, Cr^3+^ is larger than Al^3+^, 0.615 Å vs. 0.535 Å [21,22], the fact that the unit cell of LATO is larger than LCTO might suggest a small amount of Al^3+^ mixing onto the tetrahedral 8a spinel site in exchange for Li^+^ (0.76 Å) on the 16d octahedral site in the *Fd*$\bar{3}$*m* space group. Site mixing is highly unlikely for d^3^ Cr^3+^ owing to its well-known high crystal field stabilization energy in octahedral coordination [23]. Site mixing of Al onto the 8a tetrahedral sites is common in spinels and is, for example, observed in MgAl_2_O_4_ [23]. Partial occupation of the 8a tetrahedral site by the heavier Al atom relative to Li would be evidenced in the XRD pattern by an increase in the intensity of the 220 (~30° 2Θ, Cu K α radiation) and the 422 (~54° 2Θ, Cu K α radiation) peaks [24].

However, comparison of the LCTO XRD pattern versus the LATO XRD pattern (Figure 1) does not indicate any significant difference in the intensity of the peaks for the two samples, suggesting that any site mixing between Al and Li in LATO is negligible. The lack of significant site mixing is further evidenced from Rietveld structure refinement using power XRD data. Structural analysis by Rietveld refinement of XRD data was done with the Fullprof program [25].

The Rietveld refinement results are plotted with WINPLOTR program [26] in the supplementary information (Figures S1 and S2) and the atomic positions and final refinement information is contained in the supplementary information (Tables S1 and S2), for LCTO and LATO, respectively. In the starting Rietveld structural model for both LCTO and LATO, Li was placed on the 8a tetrahedral site, Li, Ti and Cr or Al were randomly distributed on the 16d octahedral site and oxygen on the 32e site of the *Fd*$\bar{3}$*m* space group. During the refinement, site mixing of Ti, Al, or Cr on the 8a tetrahedral site was explored but led to a lower goodness of fit. That is, in the fully converged refinements, the 8a octahedral site is occupied by Li and the 16d octahedral site is randomly occupied by Li, Ti, and (Cr or Al). The oxygen positional parameter (*u*), the atomic position coordinate of oxygen in the 32e site, was refined to a value of 0.26321 and 0.26340 for LCTO and LATO, respectively. The *u* values should be taken as an approximation since it is relatively difficult to locate oxygen positions through X-ray diffraction. Neutron diffraction will be needed to definitively define the oxygen position. The relative value of the positional parameter is in accordance with what is expected based on the relative sizes of Cr^3+^ and Al^3+^ [27]. If the origin of the unit cell is taken as the center of symmetry, in an ideal, cubic closed packed oxygen lattice, the oxygen parameter, *u*, has a value of 0.250. In the case of *u* = 0.250, the octahedral cation–oxygen bond length is 1.155 times longer than the tetrahedral–oxygen bond length [27]. However, the spinel oxygen parameter changes depend on the size and charge of the cations occupying the tetrahedral and octahedral sites, distorting the cubic closed packed oxygen lattice to accommodate different ions. In fact, more than 30 different ions of varying size can be accommodated in the spinel structure [27]. To accommodate large cations on the tetrahedral sites, oxygens are displaced along the [11] direction increasing the tetrahedral cation–oxygen bond length and concurrently decreasing the octahedral cation–oxygen bond length, leading to an increase in *u* and vice versa. Manipulation of the *u* parameter through changes in site occupation of octahedral sites might be a tool to optimize Li-ion conductivity in spinels.

In summary, both LCTO and LATO are single phase spinel structured materials with Li occupying 8a and 16d sites, Cr, Al and Ti randomly occupying the 16d sites in a nearly cubic close packed oxygen framework. The oxygen framework adjusts the positions of the oxygens within the unit cell to accommodate the relative difference in size of Cr versus Al.

XRD patterns of hot-pressed and Li_3_BO_3_ (LBO)-containing samples are shown in Figure 2, indicating no new peaks, i.e., no new phases, and retention of the single-phase spinel structure. LBO, a low-melting sintering aid, was added to increase density and thereby improve conductivity and mechanical properties.

LBO is not detected in the XRD pattern owing to the light elements present and the low concentration. Generally, phases at or below a few weight% are not detected and some site substitution on spinel is also a possibility. The lattice constant as a function of Li_3_BO_3_ content for hot-pressed pellets was determined by grinding the pellets to a powder, the addition of NIST-traceable Si as an internal peak position standard and Rietveld refinement of the XRD pattern. The obtained lattice constants are tabulated and plotted in the supplementary information, Table S3 and Figure S3, respectively. In the case of LATO, there is an increase in the lattice constant of hot-pressed pellets with the addition of 3% LBO to LATO from 8.3459(1) to 8.3474(1) Å. The LCTO lattice constants change in a more complicated manner. There is an initial increase in the lattice constant from 0% to 1% LBO, 8.3444(1) to 8.3456(1) Å and then a roughly linear decrease in lattice constant from 1, 1.5 and 3% LBO, 8.3456(1), 8.3450(1), 8.3449(1) Å, respectively. An increase might be attributed to substitution of additional relatively large Li onto the lattice and the decrease to substitution of the relatively small B. The evidence for site substitution by B is in agreement with the WDS analysis, which showed B distributed throughout the samples and not only at grain boundaries.

### 2.2. Microstructure

**Figure 3:**
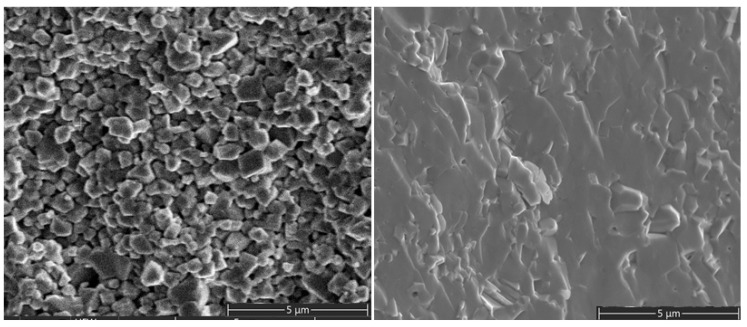
Representative SEM image of fracture surface images of hot-pressed Li_1.25_Cr_0.25_Ti_1.5_O_4_ without LBO, **left**, and with 1% LBO, **right**.

Representative micrographs of the fracture surfaces of the hot-pressed LCTO sample without LBO (left) and with 1% LBO (right) are shown in Figure 3**.** From the SEM analysis, a couple of important points are noted. First, the LBO-containing sample is very dense in agreement with the high relative density ~98%, determined from the physical dimensions, weight and the theoretical density in contrast to the ~94% density of the sample without LBO. Almost no porosity is observable in the LBO-containing sample. A high relative density is extremely important for device applications because it leads to increased mechanical strength and higher ionic conductivity.

Second, the fracture surface is very flat indicating transgranular fracture revealing high grain boundary strength, which should lead to low inter-granular ionic resistance whereas for the LBO free sample the fracture node is primarily intergranular leading to higher grain boundary resistance. Third, the average grain size observable for the LBO free sample is about 1 μm. In the LBO-containing sample, the grain size is roughly estimated to be about 2–5 μm, though the grains are difficult to distinguish.

### 2.3. Conductivity

**Figure 4:**
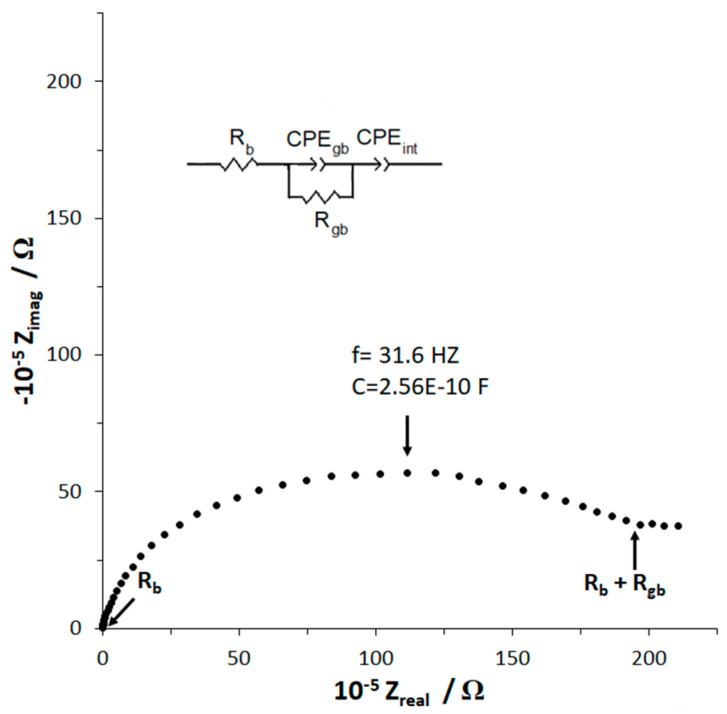
Room temperature impedance plot of hot-pressed Li_1.25_Cr_0.25_Ti_1.5_O_4_ and the equivalent circuit used to interpret the data.

The room temperature impedance plot for hot-pressed Li_1.25_Cr_0.25_Ti_1.5_O_4_ (~94% relative density) and the equivalent circuit (inset) which models the data are shown in Figure 4. In the equivalent circuit, R refers to resistance, and CPE to constant phase element. The impedance spectra shows a single semi-circle at higher frequency and starts to level off at lower frequency, which we interpret as the precursor to an upward sloping line. A fit of the data, based on this interpretation using the indicated equivalent circuit, is included in the supplementary information (Figure S4, Table S4). The equivalent circuit for this system where ionic conduction is predominant includes R_b_, bulk or intra-grain impedance, R_gb_, the grain boundary or inter-grain impedance, CPE_gb_, the grain boundary constant phase element and CPE_int_, the sample electrode interface or dual layer constant phase element which is physically attributed to charge build-up at the electrode [28,29,30]. Since we used Li-ion blocking electrodes, the shape of the curve represents a material, which is predominantly a Li-ion conductor with low electronic conductivity [28,29,30]. From Figure 4, several important points are noted.

First, the calculated value of the capacitance using the frequency at the maximum point of the semi-circle is shown on Figure 4. This capacitance, 2.56 × 10^−10^F, was calculated from C_gb_ = (2π*f*R)^−1^, using *f* = 31 Hz and R (diameter of the semi-circle) = 1.97 × 10^5^ Ω [31]. Second, this capacitance value is characteristic of a grain boundary [31] confirming the assignment of this semi-circle to a grain boundary phenomenon. The bulk impedance value, R_b_ can be taken from the Z_real_ intercept at the high frequency of the semi-circle and the total impedance, R_total_ = R_b_ + R_gb_, is taken from the Z_real_ low frequency intercept. Third, the values of R_b_ and R_gb_ and the physical dimensions of the sample are then used to determine the Li-ion conductivity. The bulk ionic conductivity of the Li_1.25_Cr_0.25_Ti_1.5_O_4_ pellet at room temperature is 1.63 × 10^−4^ S cm^−1^ and the total ionic conductivity of Li_1.25_Cr_0.25_Ti_1.5_O_4_ is 2.84 × 10^−8^ S cm^−1^. This bulk ionic conductivity is in the range of Al substituted Li_7_La_3_Zr_2_O_12_ cubic garnet solid-state electrolyte when first reported by Murugan et al. [1], however, the total ionic conductivity is three to four orders of magnitude lower than cubic garnet indicating relatively high grain boundary impedance. In fact, the ratio of grain boundary impedance to the total ionic impedance is 99.93%. We compare the ratio of grain boundary impedance to the total ionic impedance since we cannot calculate a grain boundary conductivity as the volume of the grain boundaries is unknown.

The electronic conductivity of Li_1.25_Cr_0.25_Ti_1.5_O_4_ at room temperature obtained from the steady state current found through DC polarization [32,33] is about 1.84 × 10^−8^ S cm^−1^. Thus, the ionic transport number, t_ionic_, for Li-ions in Li_1.25_Cr_0.25_Ti_1.5_O_4_ (t_ionic_ = σ_ionic_/σ_total_, where σ_total_ = σ_ionic_ + σ_electronic_; σ_ionic_ is the total ionic conductivity) is about 0.6, confirming that Li_1.25_Cr_0.25_Ti_1.5_O_4_ is an ionic conductor, yet having a significant electronic conductivity. Similarly, Li_1.25_Al_0.25_Ti_1.5_O_4_ was prepared, densified through hot-pressing and analyzed. The bulk ionic, total ionic and electronic conductivities obtained from the 97% relative density Li_1.25_Al_0.25_Ti_1.5_O_4_ pellet are 5.11 × 10^−5^, 4.08 × 10^−7^, and 9.79 × 10^−8^ S cm^−1^, respectively, a slightly lower bulk conductivity but a slightly higher bulk ionic and electronic conductivity than that of the 94% relative density LCTO pellet. The ionic transport number, t_ionic_, for Li-ions in Li_1.25_Al_0.25_Ti_1.5_O_4_ is 0.81, evidencing a higher ionic component compared to LCTO as might be expected owing to the substitution of more easily reducible Cr^3+^ compared to Al^3+^. The lower bulk conductivity in LATO relative to LCTO most likely results from the difference in lattice constant, since the Li content is similar. Similarly to LCTO, almost all ionic impedance in the LATO pellet, 99.20%, originates at grain boundaries.

**Figure 5:**
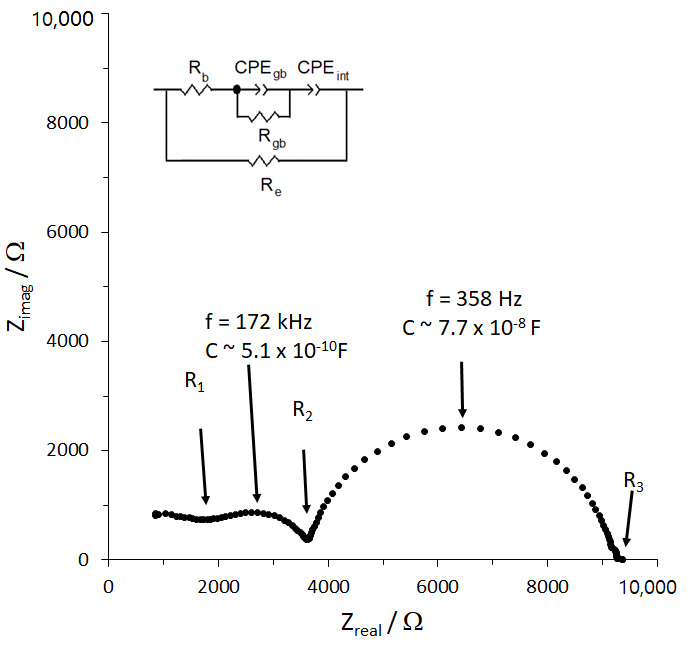
Room temperature impedance plot of hot-pressed Li_1.25_Cr_0.25_Ti_1.5_O_4_/3% LBO and the equivalent circuit used to interpret the data where R_1_ = R_e_R_b_/(R_e_ + R_b_), R_2_ =R_e_(R_b_ + R_gb_)/(R_e_ + R_b_ + R_gb_), and R_3_ =R_e_.

In order to overcome the high grain boundary impedance and to increase the density of the samples, Li_3_BO_3_ (LBO) was used as a sintering and hot-pressing aid. The room temperature impedance plot of Li_1.25_Cr_0.25_Ti_1.5_O_4_ hot-pressed with 3 wt% LBO to form a pellet of 98% relative density and the equivalent circuit (inset) which models the data are shown in Figure 5. This figure will be used to illustrate the interpretation of the EIS data for all of the LBO-containing samples. From Figure 5, several points can be made. First, the capacitance is calculated as previously described in the discussion of Figure 4 for the first two semi-circles from higher (right) to lower frequency and the values are noted on Figure 5. The calculated capacitances are characteristic of grain boundary and bulk phenomena for the higher frequency and lower frequency semicircles, respectively.

Second, the shape of the impedance plot is characteristic of an ionic conductor with electronic conductance [28], in agreement with the observation that hot-pressing with the LBO sintering aid changed the color of the samples from green (LCTO) and white (LATO) to black. Modelling the transport, therefore, requires the addition of a parallel electronic resistance, R_e_, to the ionic-conduction circuit [28,29]. The values of the resistances in the equivalent circuit, bulk ionic resistance (R_b_), grain boundary ionic resistance (R_gb_), and electronic resistance (R_e_) can be determined from the intercepts, R_1_, R_2_ and R_3_, respectively, based on the following relationships: R_1_ = R_e_R_b_/(R_e_ + R_b_), R_2_ = R_e_(R_b_ + R_gb_)/(R_e_ + R_b_ + R_gb_), and R_3_ =R_e_ [28,29]. Finally, the values of R_b_, R_ion_, and R_e_ and the physical dimensions of the sample are then used to determine the Li-ion and electronic conductivities.

For the LBO-containing samples, two pellets were analyzed for each composition. The highest total ionic conductivity was found for the Li_1.25_Cr_0.25_Ti_1.5_O_4_/1 wt. % LBO composition and is 4.17 × 10^−4^ S cm^−1^. The room temperature total Li^+^ ion conductivity measured for a pellet of Li_1.25_Cr_0.25_Ti_1.5_O_4_—1 wt.% LBO is near the range of the highest ever reported for an oxide [34]. By comparison, substituted cubic garnet Li_7_La_3_Zr_2_O_12_ has reported total Li-ion conductivity ranging from 5 × 10^−4^ to 1 × 10^−3^ S cm^−1^ [34]. The percent of ionic impedance from the grain boundary dropped to 19.31% in this sample. The DC electronic conductivity of Li_1.25_Cr_0.25_Ti_1.5_O_4_/1 wt.% LBO averaged 3.8 × 10^−4^ S cm^−1^ which means this composition may have applicability as an anolyte or catholyte where mixed electronic and ionic conductivity is important. The AC electronic conductivity value was in agreement with the DC measurement.

With the addition of 1 wt. % LBO, density increased, bulk ionic conductivity was slightly increased, total ionic conductivity increased by two to three orders of magnitude, and the electronic conductivity increased by four orders of magnitude. Thus, use of an optimal amount (~1 wt.%) of LBO might be particularly attractive to increase electronic conductivity and total ionic conductivity as a catholyte or anolyte. Data for all samples are tabulated in Table 1.

**Figure 6:**
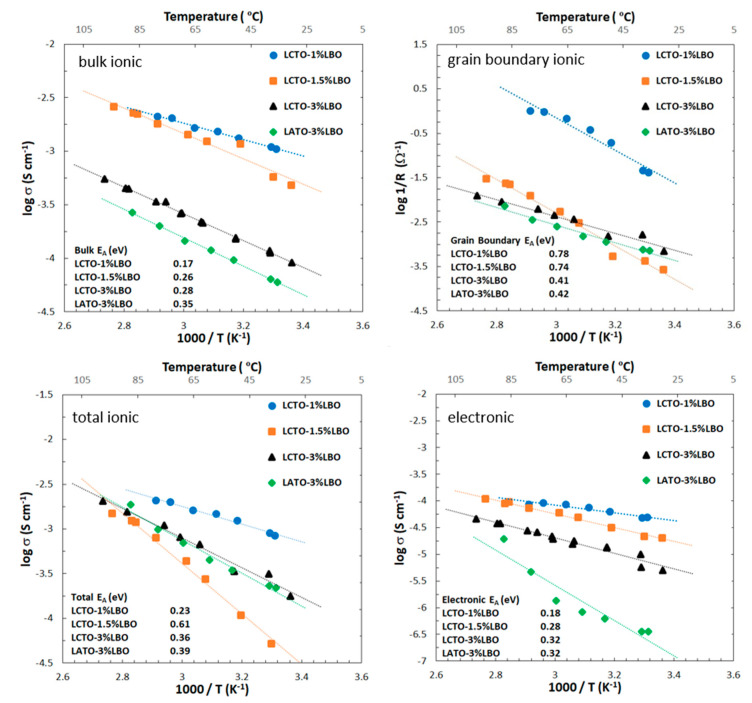
Bulk ionic, total ionic and electronic conductivities and (1/R_gb_) grain boundary resistance plots of Li_1.25_CrTi_1.5_O_4_ (LCTO) and Li_1.25_Al_0.25_Ti_1.5_O_4_ (LATO) with varied weight percent Li_3_BO_3_ (LBO) as a function of temperature. E_A_ is the activation energy. Plotting (1/R_gb_) was done since the grain boundary conductivity cannot be calculated because the grain boundary volume is unknown [35].

Temperature-dependent conductivity data collection focused on the higher conductivity LBO-containing samples. Bulk ionic, grain boundary ionic, total ionic and electronic conductivities of Li_1.25_Cr_0.25_Ti_1.5_O_4_ (LCTO) and Li_1.25_Al_0.25_Ti_1.5_O_4_ (LATO) with varied weight percent LBO are shown in Figure 6 as a function of temperature. Log (1/Rgb) versus 1/T was plotted for the grain boundary data since one cannot calculate σ_gb_ since the grain boundary volume is unknown and it only differs from plotting log(σ_gb_) versus 1/T by a constant [35]. In Figure 6, log σ is plotted as a function of 1/T in order to ease the reading of the conductivity values; however, all values of the activation energies E_A_ were calculated based on log (σT) plotted as a function of 1/T, where σ is the conductivity (S cm^−1^) and T is the temperature (K). For the 1/T vs. log (σT) plots, see the supplementary information, Figure S5. From Figure 6, several points can be made. First, all samples show fast room temperature Li-ion conductivity ranging from ~10^−4^ to ~10^−3^ S cm^−1^ and comparable electronic conductivity, suggesting applicability as mixed ionic electronic conductors and the LCTO-1% LBO composition stands out for both high ionic and electronic conductivity. Use of these materials as solid electrolytes will require the discovery of an alternate sintering aid or an alternate densification process to increase density and reduce grain boundary impedance perhaps under oxygen in order to maintain low electronic conductivity or the use of an interfacial layer such as Li_3_N [17]. However, it may find greater applicability as a catholyte or anolyte where mixed electronic ionic conductivity is desirable.

Turning attention to the bulk ionic conductivity activation energies, the values range from 0.18 to 0.28, 0.32 and 0.32, respectively for LCTO-1%LBO, LCTO-1.5%LBO, LCTO-3%LBO and LATO-3%LBO, respectively. The bulk activation energies are close to what is reported for other fast Li-ion conductors indicating fast Li-ion mobility [34,36,37,38,39].

The especially low bulk activation energy of LCTO-1% helps to explain the very high Li-ion conductivity of this composition. These bulk activation energies are lower than the activation energy of 0.35 eV reported from Li NMR line broadening experiments on spinel-structured, Li-doped MgAl_2_O_4_ [12]. The grain boundary activation energies range from 0.78 to 0.74, 0.41 and 0.42 eV for LCTO-1%LBO, LCTO-1.5%LBO, LCTO-3%LBO and LATO-3%LBO, respectively, suggesting that the addition of a higher concentration of LBO has a strong effect to lower the activation energy for ionic conductivity at the grain boundary. However, looking at the total ionic conductivities shows a clear superior performance for the LCTO-1%LBO sample, its total conductivity predominantly controlled by its higher bulk ionic mobility despite higher activation energy at its grain boundaries. The total ionic conductivity activation energies range from 0.23 to 0.61, 0.36, 0.39 eV for LCTO-1%LBO, LCTO-1.5%LBO, LCTO-3%LBO and LATO-3%LBO, respectively. The addition of excess LBO, >1%, negatively affects the total Li-ion conductivity although at 3% LBO the grain boundary ionic activation energy is considerably lower. The LCTO-1.5%LBO sample appears to be an outlier, as one would expect it to fall between the 1% and the 3% LBO samples. The electronic conductivities activation energies range from 0.18 to 0.28, 0.32, and 0.32 eV for LCTO-1%LBO, LCTO-1.5%LBO, LCTO-3%LBO and LATO-3%LBO, respectively. It is observed that at a low level of LBO the electronic conductivity is highest and as more LBO is added the electronic conductivity decreases. Overall, it appears that the 1%LBO sample has the maximum electronic and ionic conductivity. It might be that the higher electronic conductivity improves the ionic conductivity owing to an enhancement effect of the transport of two species [40,41,42]. That is, as a Li-ion hops, in order to preserve charge neutrality both species must move at the same speed which means the faster moving electron is slowed down while the speed of the slower moving Li^+^ ion is increased.

### 2.4. Electrochemical Properties of Solid Solutions of LiNi_0.5_Mn_1.5_O_4_ and “Li_1.25_Cr_0.25_Mn_1.5_O_4_”

Demonstration of the practical applicability of Li_1.25_(Cr,Al)_0.25_Ti_1.5_O_4_ spinels as solid electrolyte to function as a separator will require finding a new densification aid that does not lead to high electronic conductivity. Subsequently, if such a densification aid is found, we will study the decomposition window of the electrolyte and build lithium symmetric cells. However, the use of the LBO densification aid leads to significant electronic conductivity, therefore this suggests that the most likely application of LBO-spinel composites is as catholyte or anolyte. Furthermore, the unique feature of demonstrating fast Li-ion conduction in spinel structured solids is the possibility to demonstrate that an electrochemically active solid solution can be formed from a solid solution with a spinel structured electrode material. A liquid based cell was used as a simple way to demonstrate this principle. Demonstration in a solid-state cell will require optimization of multiple properties and will be part of a future study.

**Figure 7:**
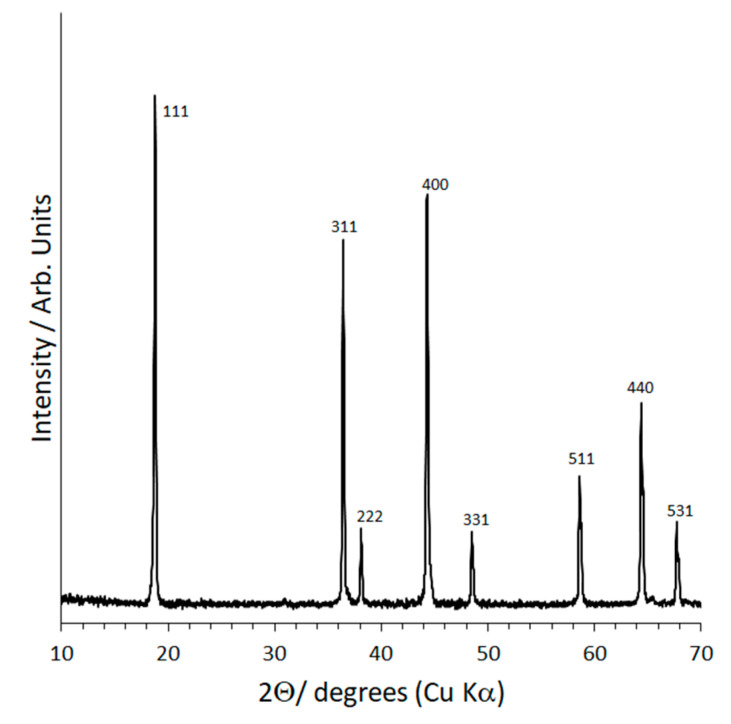
XRD pattern of 0.3 [Li_1.25_Cr_0.25_Mn_1.5_O_4_]: 0.7 [LiNi_0.5_Mn_1.5_O_4_] solid solution formed at 850 °C (nominal composition: Li_1.025_Cr_0.025_Ni_0.45_Mn_1.5_O_4_). XRD peaks are indexed to the *Fd*$\bar{3}$*m* spinel structure.

Since LiNi_0.5_Mn_1.5_O_4_ is known to form high-impedance phases when formed into a composite with Li_7_La_3_Zr_2_O_12_ garnet and other well-studied solid electrolytes, it was decided that it would be a good test case with practical applicability. We found that LCTO and LATO form single-phase, spinel-structured solid solutions with LiNi_0.5_Mn_1.5_O_4_ and also Li_4_Ti_5_O_12._ However, as a first test-case, Mn was substituted for Ti in the solid electrolyte component owing to the known deleterious effect of significant Ti substitution for Mn in LiNi_0.5_Mn_1.5_O_4_ [43]. This substitution can be done to produce a Li-stuffed spinel of composition Li_1.075_Cr_0.075_Ni_0.35_Mn_1.5_O_4_, though the Li_1.25_Cr_0.25_Mn_1.5_O_4_ end component could not be successfully synthesized owing to the formation of Li_2_MnO_3_ impurity. Follow-up studies will look in detail at the electrochemical properties, the mixed electronic-ionic conductivities, spinel phase stability, and electrochemical stability windows of the vast solid-solution range of materials in the families Li_1.25_(Cr,Al)_0.25_(Mn,Ti)_1.5_)O_4_: LiNi_0.5_Mn_1.5_O_4_ and Li_1.25_(Cr,Al)_0.25_(Mn,Ti)_1.5_)O_4_: Li_4_Ti_5_O_12_). The range of solid solution formation will be part of this future study. For the current study, we chose a 3:7 ratio to compare to a typical amount of porosity (30%) in a standard cell. In other words, the “electrolyte” component of the solid solution was set at 30%. This was a first estimate and is not yet optimized. The XRD pattern of nominal composition 0.3[Li_1.25_Cr_0.25_Mn_1.5_O_4_] 0.7[LiNi_0.5_Mn_1.5_O_4_], i.e., Li_1.075_Cr_0.075_Ni_0.35_Mn_1.5_O_4_ composition is shown in Figure 7. The lattice constant was refined to 8.1704(1) Å, which is comparable to LiNi_0.5_Mn_1.5_O_4_ (8.1785 Å) [44] the predominant component of the solid solution. The slight decrease in the lattice constant is to be expected based on the slightly smaller average size of Li^+^, Cr^3+^ (0.76 Å, 0.615 Å, averaging 0.6875 Å) compared to Ni^2+^ (0.69Å) in octahedral coordination [21,22]. The pattern is indexed to the cubic spinel structure, space group, *Fd*$\bar{3}$*m*, indicating a single-phase composition of spinel structure. Structural analysis by Rietveld refinement of XRD data was done with the Fullprof program. The Rietveld refinement result is plotted in the supplementary information, Figure S6, and the atomic positions and final refinement information is contained in the supplementary information, Table S5. In the Rietveld structural model for Li_1.075_Cr_0.075_Ni_0.35_Mn_1.5_O_4_, the 8a tetrahedral site is occupied by Li, the 16d octahedral site contains Li, Mn, Cr and Ni and oxygen occupies the 32e site of the *Fd*$\bar{3}$*m* space group. During the refinement, site mixing of Cr, Ni or Mn on the 8a tetrahedral site was explored but did not improve the fit. That is, in the fully converged refinements, Li fully occupies the 8a tetrahedral and the 16d octahedral is randomly occupied by Li, Mn, Ni and Cr.

**Figure 8:**
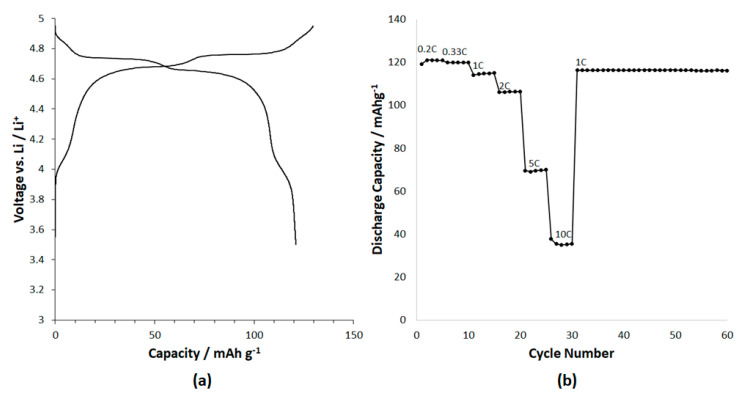
(**a**) Electrochemical charge and discharge curve of 30% Li_1.25_Cr_0.25_Mn_1.5_O_4_ and 70% LiNi_0.5_Mn_1.5_O_4_ solid solution formed at 850 °C (nominal composition: Li_1.025_Cr_0.025_Ni_0.45_Mn_1.5_O_4_) (**b**) Discharge capacity of Li_1.075_Cr_0.075_Ni_0.35_Mn_1.5_O_4_ as function of charge and discharge rate at a loading of about 6 mg cm^−1^ (~ 0.7 mAh cm^−1^). Symmetrical charge and discharge rate indicated on the figure were varied each five cycles (cycles 1–30) and fixed at 1C for cycles 31–60.

The oxygen positional parameter (*u*), the atomic position coordinates of oxygen in the 32e site refined to a value of 0.26266, in accordance with expectation from the ionic radii of the tetrahedrally and octahedrally coordinated cations [27]. In order to demonstrate expediently that the Li_1.025_Cr_0.025_Ni_0.45_Mn_1.5_O_4_ composition is electrochemically active, a liquid electrolyte-based cell was built. In the future, as composition, LBO content, microstructure and the ability to fabricate a thin, dense catholyte is optimized and developed, the concept will be tested in a solid-state configuration. The electrochemical discharge curve at 0.2C charge and discharge rate of the nominal composition 0.3[Li_1.25_Cr_0.25_Mn_1.5_O_4_] 0.7[LiNi_0.5_Mn_1.5_O_4_], i.e., Li_1.075_Cr_0.075_Ni_0.35_Mn_1.5_O_4_ composition is shown in Figure 8a. A discharge capacity ~120 mAh g^−1^ is observed. Assuming electrochemical activity based on Ni^2+^/Ni^3+^, Ni^3+^/Ni^4+^ and Cr^3+^/Cr^4+^ couples yields a theoretical capacity of about 116 mAh g^−1^ for Li_1.075_Cr_0.075_Ni_0.35_Mn_1.5_O_4_. The theoretical discharge capacity is observed at >4.6 V. Additional capacity, beyond the theoretical, can be attributed to the Mn^3+^/Mn^4+^ couple observed as a shoulder around 4 V. The good rate performance resulting from high Li^+^ ionic conductivity and electronic conductivity is shown in Figure 8b. The charge and discharge rates are varied from 0.2 to 10 C, with the charge and discharge rates remaining equal for each individual cycle. Additionally, the material shows good cycle life and no damage from the high rate of charge and discharge is evidenced as the capacity returns to 120 mAh g^−1^ for 1 C after charging and discharging at 10 C rate. Further measurements and optimization of the mixed ionic electronic conductivity through composition and electrode design will be needed to fully realize the maximum rate performance. Further studies will be needed to characterize the conductivity of the solid electrolyte–solid cathode solid solutions as a function of composition and with the addition of LBO to improve grain boundary conductivity. This preliminary result confirms the formation of an electrochemically active solid solution.

### 2.5. Composition

The elemental composition of the Li_1.25_Cr_0.25_Ti_1.5_O_4_ and Li_1.25_Al_0.25_Ti_1.5_O_4_ powders as obtained from ICP is tabulated in the supplementary information (Table S6) and is in excellent agreement with the nominal composition. In the LBO-containing samples, the semi-quantitative distribution of boron (B) was probed by wavelength dispersive spectroscopy (WDS) analysis of the cross-section of two representative pellets with 1 and 3% LBO. Over multiple spots spanning the length thickness of the pellet, B was spread through the whole sample, suggesting that the B is not concentrated at the grain boundaries. Since it was not possible to differentiate the grain boundary from the bulk during the data collection, we cannot definitively locate B since there is some chance that the measurement were done only on grain boundary or only bulk spots, the spot size is rather large (5 μ) and the error in measurement (±2% based on counting statistics) is on the order of the concentration (1–3% LBO). However, the analysis of XRD data and the changes in both bulk and grain boundary conductivity described later all support the conclusion that B is distributed throughout the sample. The B distribution from WDS is tabulated in the supplementary information (Table S7) and the images of the pellets used for determination of B distribution are shown in Figure S7.

## 3. Discussion

The introduction of Li on multiple sites has been previously demonstrated to be a successful strategy to attain higher Li^+^ ion conductivity [1,15,16]. As an example, in garnet structured Li_7_La_3_Zr_2_O_12_, one of the most well-known oxide-based Li^+^ conductors (10^−3^ to 10^−4^ S cm^−1^ at room temperature) Li^+^ is sited on both tetrahedral (24d) and octahedral (96h) sites and the occupancy ratio between the sites is critical to optimization of the conductivity [45]. Similarly, the bulk ionic conductivity of LiTi_2_(PO_4_)_3_ jumps by 3 orders of magnitude when additional Li occupies another crystallographic site as a result of partial substitution of Ti^4+^ by Al^3+^ to form Li_1.3_Ti_0.7_Al_0.3_(PO_4_)_3_ [46,47]. By contrast, LiNi_0.5_Ge_1.5_O_4_ of modest Li^+^ conductivity (10^−8^ S cm^−1^ at 63 °C) has Li only on the tetrahedral 8c site and Ni and Ge ordered 1:3 over the 4b and 12d octahedral sites of the ordered P_4_32 spinel.

The high Li-ion conductivity observed in LCTO and LATO can be compared to the *Fd*$\bar{3}$*m* spinel structured anode Li_4_Ti_5_O_12_ which has been reported to be a rather poor Li ion conductor [48,49,50] based on Li-NMR studies. This Li-NMR study is in conflict with the fact that Li_4_Ti_5_O_12_ is a high-rate anode material [51,52], and that discrepancy was attributed by the authors to a fast ion conducting interphase which forms immediately upon Li insertion. Another study based on muon spin spectroscopy of Li_4_Ti_5_O_12_ and LiTi_2_O_4_ spinels come to a different conclusion. Their muon spectroscopy results point to very mobile Li ions in Li_4_Ti_5_O_12_ and lower activation energy for Li motion relative to LiTi_2_O_4_ [53]_._ In Li_4_Ti_5_O_12_, the 8a tetrahedral site is occupied by Li as in LCTO and LATO, and the 16d octahedral site is occupied by Li and Ti in ratio of 1:5. In LiTi_2_O_4_, Li only occupies the 8a tetrahedral site and Ti completely occupies the 16d octahedral site. This is therefore further evidence of the positive effect of multiple site occupation on Li mobility.

Integration of solid electrolytes into electrodes has been problematic thus far in published studies of solid-state batteries. Density functional theory (DFT) computational studies have shown reactivity of cubic garnet structured Li_7_La_3_Zr_2_O_7_ and common Li-ion cathode materials [54,55] and experimental studies have shown the reactivity of LiNi_0.5_Mn_1.5_O_4_ cathode with LLZO during electrochemical cycling [56]. Furthermore, LiCoO_2_ forms unfavorable interfaces during densification and requires a LiNbO_3_ coating to reduce reactivity [57]. Solid solutions of Li_1.25_(Al,Cr)_0.25_(Ti,Mn)_1.5_O_4_ with known electrode materials such as LiNi_0.5_Mn_1.5_O_4_, e.g., x[LiNi_0.5_Mn_1.5_O_4_] 1-x[Li_1.25_(Al,Cr)_0.25_(Ti,Mn)_1.5_O_4_] (0 < x < 1) with grain boundary engineering through use of LBO or other sintering aids offer an alternative, simpler route since the solid electrolyte-solid electrode interface is eliminated and the LBO increases the electronic conductivity, which is needed for use as an electrode. This should lead to increased power owing to improved Li-ion and electronic conductivity within the electrode and the solid solutions could be used as part of an all solid-state battery with a garnet based separator and Li metal anode. If a new sintering aid is found that does not lead to electronic conductivity, these spinel electrolytes might be used in a fully spinel structured all-solid battery as envisioned by Thackeray and Goodenough [11] or with an interfacial layer such as Li_3_N [17] separating it from a Li metal or carbon anode. The LiNi_0.5_Mn_1.5_O_4_ spinel is particularly attractive for solid-state application owing to its high energy storage density, high voltage (~4.7 V), use of abundant chemicals, small lattice change during charge and discharge and high Li diffusivity throughout the range of Li composition [58].

## 4. Materials and Methods

### 4.1. Powder Preparation

The compound Li_1.25_Cr_0.25_Ti_1.5_O_4_ was prepared by solid-state reaction from a stoichiometric ratio of TiO_2_ (Sigma-Aldrich, Saint Louis, MO, USA) and Cr_2_O_3_ (Alfa Aesar, Ward Hill, MA, USA) and a 3% stoichiometric excess of Li_2_CO_3_ (Alfa Aesar, Ward Hill, MA, USA) to counteract volatilization of Li. The precursors were ground by hand using a mortar and pestle, then the fine, mixed powder was heated in an uncovered alumina crucible at 10 °C per minute to 600 °C and held at this temperature for 10 h in air and allowed to furnace cool. The resulting powder was reground and pelletized using a SPEX Sample Prep 13 mm diameter pellet die (Spex Sampleprep LLC, Metuchen, NJ, USA) and Carver laboratory press (Fred S. Carver Company, Wabash, IN, USA) at a load of about 2300 kg. The pellet was placed in a covered alumina crucible and heated at 10 to 850 °C and held at this temperature for 24 h in air and then allowed to furnace cool. Li_1.25_Al_0.25_Ti_1.5_O_4_ and Li_1.25_Cr_0.25_Mn_1.5_O_4_ was prepared similarly substituting Al_2_O_3_ (Alfa Aesar, Ward Hill, MA, USA) for Cr_2_O_3_ and MnCO_3_ for Al_2_O_3_ or Cr_2_O_3_, respectively. Li_3_BO_3_ (LBO) was prepared from a stoichiometric mixture of Li_2_CO_3_ (Alfa Aesar, Ward Hill, MA, USA) and H_3_BO_3_ (Alfa Aesar, Ward Hill, MA, USA)_._ The starting mixture was ground in a mortar and pestle and heated at 600 °C in air for 4 h. Solid solutions of the solid electrolyte and the LNMO cathode were prepared through an aqueous solution based route from a mixture Li_2_CO_3_ (Alfa Aesar, Ward Hill, MA, USA), MnCO_3_ (Alfa Aesar, Ward Hill, MA, USA), Ni(OH)_2_ (Alfa Aesar, Ward Hill, MA, USA), Cr(NO_3_)_3_.9H_2_O (Alfa Aesar, Ward Hill, MA, USA) precursors dissolved in a citric acid (Sigma-Aldrich, Saint Louis, MO, USA)/nitric acid (Sigma-Aldrich, Saint Louis, MO, USA) solution. As an example, Li_1.075_Cr_0.075_Ni_0.35_Mn_1.5_O_4_ was prepared from 0.2659 g Li_2_CO_3_ (3% excess) 1.1208 g MnCO_3_, 0.2109 g Ni(OH)_2_, and 0.2401 g Cr(NO_3_)_3_.9H_2_O, 1 g citric acid and 6 g concentrated HNO_3_ diluted to 30 mL with H_2_O. The clear green solution obtained from heating the mixture was evaporated to dryness and then heated under air in a Lindbergh furnace at 10 °C per minute to 450 °C, held for 3 h, heated at 10 °C per minute to 850 °C, held for 6 h, then furnace cooled (Lindbergh/MPH, Riverside, MI, USA).

### 4.2. Consolidation of Samples for Conductivity Measurements

Sintering to obtain dense pellets was attempted in air at 850 °C. Sintering pure Li_1.25_Cr_0.25_Ti_1.5_O_4_ led to pellets of low density (~60–70% relative density) and the temperature could not be increased owing to the formation of a ramsdellite-structured phase at higher temperature. The addition of LBO led to much higher density pellets (~80–85%). LBO was chosen owing to the fact that its melting point of ~700 °C is below the 850 °C consolidation temperature, so that it can act as a liquid-phase sintering aid and also because LBO itself has moderate ionic conductivity which might enhance conduction at the grain boundaries unlike other typical sintering additives such as LiF which are poor Li-ion conductors and have melting point above the temperature at which the samples are converted from the spinel to the ramsdellite structure. A similar spinel to ramsdellite phase transformation upon heating has been previously documented for LiTi_2_O_4_ [59]. Dense discs (>90%) were prepared by rapid induction hot-pressing (custom built by University of Michigan, Ann Arbor, MI, USA) without (93–97%) and with LBO (near 100%). For the higher conducting Li_1.25_Cr_0.25_Ti_1.5_O_4_, three different amounts of LBO were tested (1, 1.5 and 3 wt. %) in an attempt to optimize conductivity. For the Li_1.25_Al_0.25_Ti_1.5_O_4_, a 3 wt. % LBO-containing hot-pressed sample was prepared based on sintering studies to increase density. The powders were densified at 850 °C at 40 MPa for 40 min under Ar using a rapid induction hot-pressing technique. The spinel powders were hot-pressed in a graphite die. During the hot-pressing, the die is contained in a stream of Ar, creating a reducing atmosphere. After, densification in the presence of LBO, the pellets changed to a black color. In the absence of LBO, no color change was observed. Attempts to oxidize reduced LBO-containing samples at 850 °C in air, in the presence of mother power to reduce Li loss by heating under air, were unsuccessful. The bulk density of the sample was determined from the weight and physical dimensions. The relative density values were estimated by dividing the measured density by the theoretical crystal density based on the spinel structure and the measured lattice constants. The presence of 1–3 wt.% lower density LBO (2.16 g cm^−3^ versus 3.48 and 3.61 g cm^−3^ for LATO and LCTO, respectively) was used to calculate a theoretical density based on the rule of mixtures and the relative density of each sample was then calculated. This can vary from actual by 1–2% based on whether LBO is incorporated into spinel or present as a distinct separate phase.

### 4.3. X-ray Diffraction

X-ray diffraction (Cu Kα radiation, Rigaku Miniflex 600, D/teX Ultra silicon strip detector, Rigaku Americas Inc., The Woodlands, TX, USA) was used to characterize the phase purity of the powders and the material after hot-pressing. To determine phase purity and for Rietveld structural analysis [60], data were collected from 10–90° 2θ at 0.02° increments at 4° per minute. Lattice constants were calculated from Rietveld Refinement of an X-ray diffraction pattern collected for the sample mixed with a NIST traceable Si internal peak position standard. Data were collected from 10–90° 2θ at 0.02° increments at 4° per minute. Rietveld refinements of XRD data were carried out in the *Fd*$\bar{3}$*m* space group with Li in 8a sites and Li, Cr or Al and Ti or Mn randomly distributed in 16d sites, occupancies fixed to the starting composition.

### 4.4. Elemental Analysis

Elemental analysis of the powders was performed at Galbraith Laboratories (Galbraith Laboratories, Inc., Knoxville, TN, USA) using Inductively Coupled Plasma-Optical Emission Spectroscopy (ICP-OES). Manually fractured, hot-pressed pellet cross-sections containing Li_3_BO_3_ were analyzed for boron concentrations via WDS using a JEOL JXA-8900 Electron Probe Microanalyzer (EPMA, Jeol USA, Inc., Peabody, MA, USA) in the Advanced Imaging and Microscopy Laboratory (AIMLab) at the University of Maryland. Analyses were conducted with a beam current of 50 nA and accelerating voltage of 15 kV. The beam diameter was 5 microns. Boron K-alpha x-rays were observed using an LDEB (Mo/B_4_C layered synthetic microstructure) analytical crystal. Raw counts were corrected using a ZAF (Z, atomic number, A, absorption, F, fluorescence) algorithm.

### 4.5. Microstructure

Hot-pressed pellets were manually fractured for cross-sectional microstructural analysis. Cross-sectional analysis was conducted using Thermo Fisher Helios (Thermo Fisher Scientific, Waltham, MA, USA) and an FEI Quanta 200F scanning electron microscopes under a 5kV accelerating voltage (FEI Company, Hillsboro, OR, USA).

### 4.6. Conductivity

The temperature-dependent ionic conductivity was determined from AC impedance measurements with a Bio-logic VMP300 (Bio-logic USA, Knoxville, TN, USA) (applied frequency range 0.1 Hz to 7 MHz) and/or a Solartron Modulab (Ametek Scientific Instruments, Oak Ridge, TN, USA) (0.1 Hz–300 kHz) with an amplitude of 10–100 mV. Ni was sputtered on the top and bottom of the hot-pressed discs to serve as Li—blocking electrode. The equivalent circuit was modelled and each data set was normalized to the geometric dimensions of the disc to determine the Li-ion conductivity. The Li-ion conduction activation energies were determined from the Arrhenius plot of the relationship of the ionic conductivities to temperature in the range of ~298K to 373K.

The electronic conductivity at room temperature was measured using DC polarization measurements at a voltage of 2 V (Solartron Modulab, Ametek Scientific Instruments, Oak Ridge, TN, USA). The steady-state current and applied voltage were used to determine the resistance, which was converted to the electronic conductivity using the specimen dimensions. Electronic conductivity was also estimated from the AC impedance data.

### 4.7. Electrochemical Measurements

Solid solution electrodes of composition Li_1.25_Cr_0.25_Mn_1.5_O_4_:LiNi_0.5_Mn_1.5_O_4_ of 3:7 mole ratio (Li_1.075_Cr_0.075_Ni_0.35_Mn_1.5_O_4_) were mixed with carbon and PVDF in an NMP slurry to produce an 80:15:5 composite coating of the active: carbon black: PVDF on an Al foil current collector. The active loading was ~6 mg/cm^3^. The C rate was based on a capacity of 147 mAh g^−1^ for LiNi_0.5_Mn_1.5_O_4_. Coin cells (Hohsen, Al clad, Pred Materials, New York, NY, USA) were fabricated using an electrolyte 1 M LiPF_6_ dissolved in EC:EMC 1:1 (weight ratio, Sigma-Aldrich, Saint Louis, MO, USA) and 2% tris (trimethylsilyl) phosphate (TCI Americas, Portland, OR, USA) an electrolyte stabilizing additive for use at high voltage [61] and Li foil (Johnson Matthey, Alpharetta, GA, USA) as anode. The electrochemical data was collected on a Maccor 4000 Battery cycler (Maccor Inc., Tulsa, OK, USA) The charge and discharge rates were equal for each charge/discharge cycle and charge and discharge rates were varied each five cycles (cycles 1–30) in order from 0.2C, 0.33C, 1C, 2C, 5C to 10C and then fixed at 1C for cycles 31–60.

## 5. Conclusions

Herein, we report the synthesis and the fast Li-ion conductivity of the spinel structured Li_1.25_(Al or Cr)_0.25_(Ti or Mn)_1.5_O_4_ and a solid solution with the LiNi_0.5_Mn_1.5_O_4_ high voltage positive electrode as examples of a large class of fast Li-ion conducting potential electrolytes and cathodes based on the spinel structure. Bulk and total ionic conductivities for 1% LBO LCTO of 6.8 × 10^−4^ and 4.2 × 10^−4^ S cm^−1^, respectively, is comparable to that of the first reported bulk and total conductivities of garnet structured Al substituted Li_7_La_3_Zr_2_O_12_ [1], 4.9 × 10^−4^ and 5.1 × 10^−4^ S cm^−1^, respectively. Li is located on both octahedral and tetrahedral sites to form a fast 3D Li^+^ ion conduction pathway in Li_1.25_(Al,Cr)_0.25_(Ti,Mn)_1.5_O_4_, potentially enabling the all-solid all-spinel-structured battery concept with Li_4_Ti_5_O_12_ spinel structured anode and LiMn_2_O_4_ or LiNi_0.5_Mn_1.5_O_4_ spinel structured cathode. Significant electronic conductivity of Cr-containing samples points towards application as a catholyte or anolyte in a solid solution with known spinel structured electrode materials. Sintering with LBO leads to a highly dense mixed ionic, electronic conductor which may have application as a catholyte or a coating layer to form an artificial solid electrolyte interface to reduce reactivity with electrolytes. Electrochemical activity in liquid electrolyte-containing cells has been demonstrated for solid solutions of Li_1.25_Cr_0.25_Mn_1.5_O_4_ and LiNi_0.5_Mn_1.5_O_4_ with discharge capacity of near or greater than the theoretical capacity of LiNi_0.5_Mn_1.5_O_4_ demonstrating the concept of a spinel catholyte and a spinel cathode reacted to form a single-phase solid solution of spinel structure. This is a small step towards demonstrating their potential applications as catholyte or anolyte in a fully solid-state electrode.

## Figures and Tables

**Table 1:** Average room temperature (298 K) bulk ionic, σ_bulk_, grain boundary ionic impedance as percentage of total impedance, Z_gb_/Z_tot_, total ionic conductivity, σ_ion_, electronic conductivity, σ_elec_ and relative density, D, of Li_1.25_CrTi_1.5_O_4_ (LCTO) and Li_1.25_Al_0.25_Ti_1.5_O_4_ (LATO) solid electrolytes with and without Li_3_BO_3_ (LBO) sintering aid.

Sample	σ_bulk_ (S cm^−1^)	Z_gb_/Z_tot_ (%)	σ_ion_ (S cm^−1^)	σ_elec_ (S cm^−1^)	D (%)
LCTO	1.63 × 10^−4^	99.9	1.19 × 10^−7^	1.84 × 10^−8^	94
LATO	5.11 × 10^−5^	99.2	4.08 × 10^−7^	9.79 × 10^−8^	97
LCTO/1% LBO	6.77 × 10^−4^	19.3	4.17 × 10^−4^	3.76 × 10^−4^	98
LCTO/1.5% LBO	4.01 × 10^−4^	94.0	1.96 × 10^−4^	1.78 × 10^−4^	99
LCTO/3% LBO	8.75 × 10^−5^	51.9	5.32 × 10^−5^	4.06 × 10^−5^	97
LATO/3% LBO	5.00 × 10^−5^	27.4	1.78 × 10^−5^	1.20 × 10^−7^	99

## Supplementary Materials

The following are available online; Figure S1: Rietveld fit of Li_1.25_Cr_0.25_Ti_1.5_O_4_; Figure S2: Rietveld fit of Li_1.25_Al_0.25_Ti_1.5_O_4_; Figure S3: Lattice constants of LCTO and LATO pellets as a function of LBO content; Figure S4: Fit of LCTO EIS data to equivalent circuit; Figure S5: Log (σT) or Log(T/R_gb_) plotted as a function of 1/T used for determination of bulk ionic, grain boundary ionic, total ionic and electronic activation energies of LCTO and (LATO) with varied weight percent of LBO as a function of temperature; Figure S6: Rietveld fit of Li_1.075_Cr_0.075_Ni_0.35_Mn_1.5_O_4_; Figure S7: SEM image of pellets used for analysis of B distribution; Table S1: Refined structural model of LCTO; Table S2: Refined structural model of LATO; Table S3: Lattice constants of LCTO and LATO as a function of LBO content; Table S4: Equivalent circuit elements and value for fit of LCTO EIS data; Table S5: Refined structural model of Li_1.075_Cr_0.075_Ni_0.35_Mn_1.5_O_4_; Table S6: Elemental analysis of LCTO and LATO; Table S7: Boron distribution in LCTO pellets.

## Sample Availability

Samples of the compounds are available from the authors through collaborative agreement.
