# Supplementary material for: Fast Li-Ion Conduction in Spinel-Structured Solids
Source: Molecules. 2021 Apr 30;26(9):2625. doi: 10.3390/molecules26092625 (PMC8124195; doi:10.3390/molecules26092625)
Supplement: Supplementary file 1 [file molecules-26-02625-s001.zip › molecules-1169367-supplementary.pdf]

molecules

Fast Li-ion conduction in Spinel-Structured Solids

Jan L. Allen[1*], Bria A. Crear[2], Rishav Choudhury[3], Michael J. Wang[3], Dat T. Tran[1], Lin Ma[1], Philip M. Piccoli[4], Jeff Sakamoto[5], and Jeff Wolfenstine[6]

[1]US Army Research Laboratory, Sensors & Electron Devices Directorate, Adelphi, MD 20783, USA; jan.l.allen8.civ@mail.mil (J.L.A.); dat.t.tran4.civ@mail.mil (D.T.T.); liam.l.ma.civ@outlook.com (L.M.)
[2]Howard University; Department of Chemistry, Washington, D.C. 20059, USA; bria.a.crear@gmail.com
[3]University of Michigan, Department of Materials Science and Engineering, Ann Arbor, MI 48109 USA; rishavc@umich.edu (R.C.); micwan@umich.edu (M.W.) [4]University of Maryland, Department of Geology, College Park, MD 20742 USA; piccoli@umd.edu [5]University of Michigan, Departments of Materials Science and Engineering, Mechanical Engineering, and Macromolecular Science and Engineering, Ann Arbor, MI 48109 USA; jeffsaka@umich.edu (J.S.) [6]Solid Ionic Consulting, 9223 Matthews Ave, Seattle, WA 98115 USA; jeffyspeak@outlook.com
* Correspondence: Jan L. Allen, jan.l.allen8.civ@mail.mil;

Supplementary Material

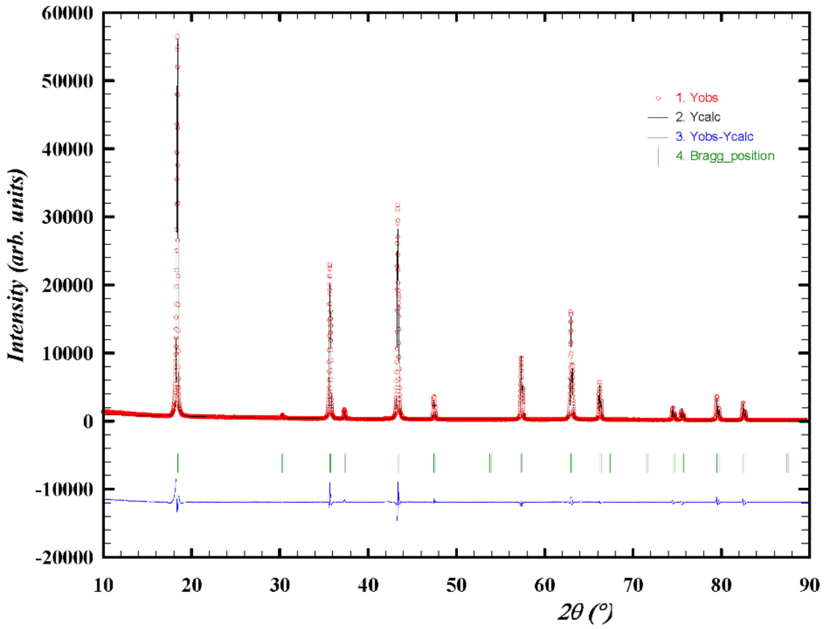

Figure S1. Rietveld fit of $Li_{1.25}Cr_{0.25}Ti_{1.5}O_4$ (LCTO) where red dots are observed points, continuous black line is calculated profile and the positions of Bragg reflections (green) and the observed intensity (Yobs) minus the calculated intensity (Ycalc) difference (blue) are shown at the bottom.

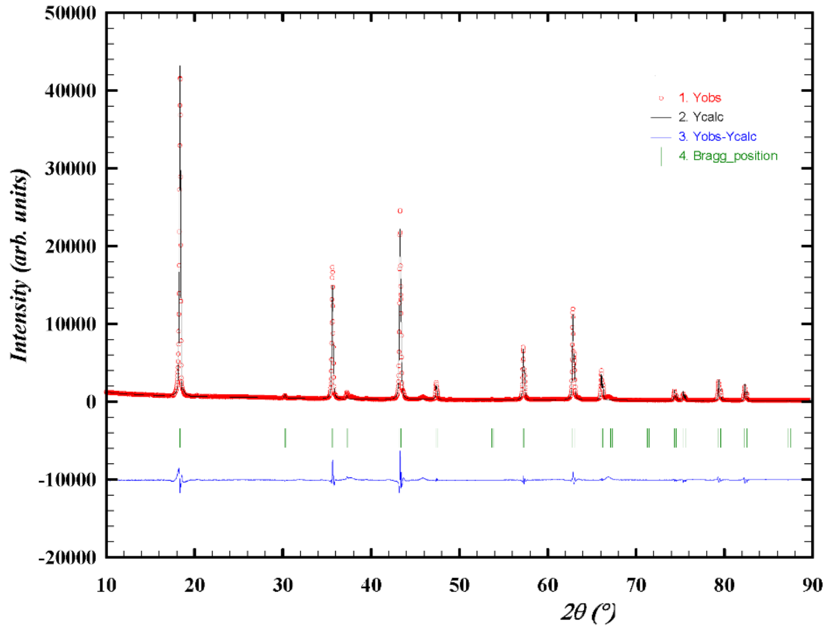

Figure S2. Rietveld fit of $Li_{1.25}Al_{0.25}Ti_{1.5}O_4$ (LATO) where red dots are observed points, continuous black line is calculated profile and the positions of Bragg reflections (green) and the observed intensity (Yobs) minus the calculated intensity (Ycalc) difference (blue) are shown at the bottom.

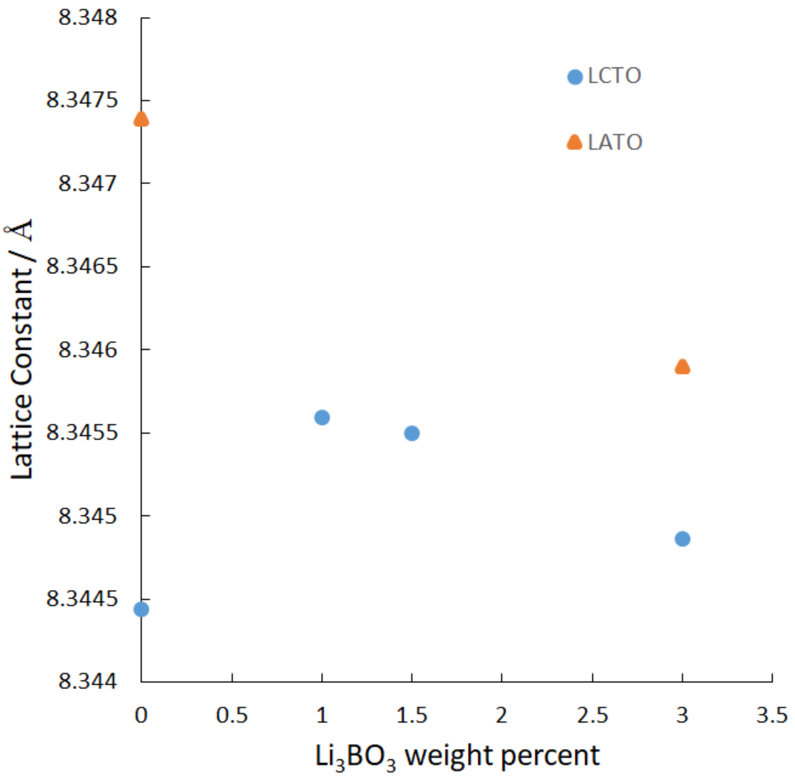

Figure S3. Lattice constants of $Li_{1.25}Cr_{0.25}Ti_{1.5}O_4$ (LCTO, blue circles) and $Li_{1.25}Al_{0.25}Ti_{1.5}O_4$ (LATO, orange triangles) pellets as a function of LBO content.

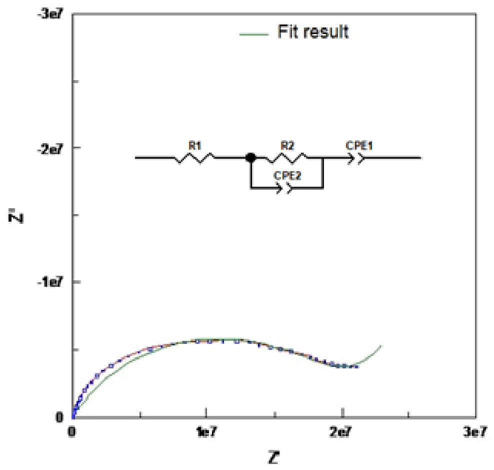

Figure S4: Fit of LCTO EIS data to indicated equivalent circuit.

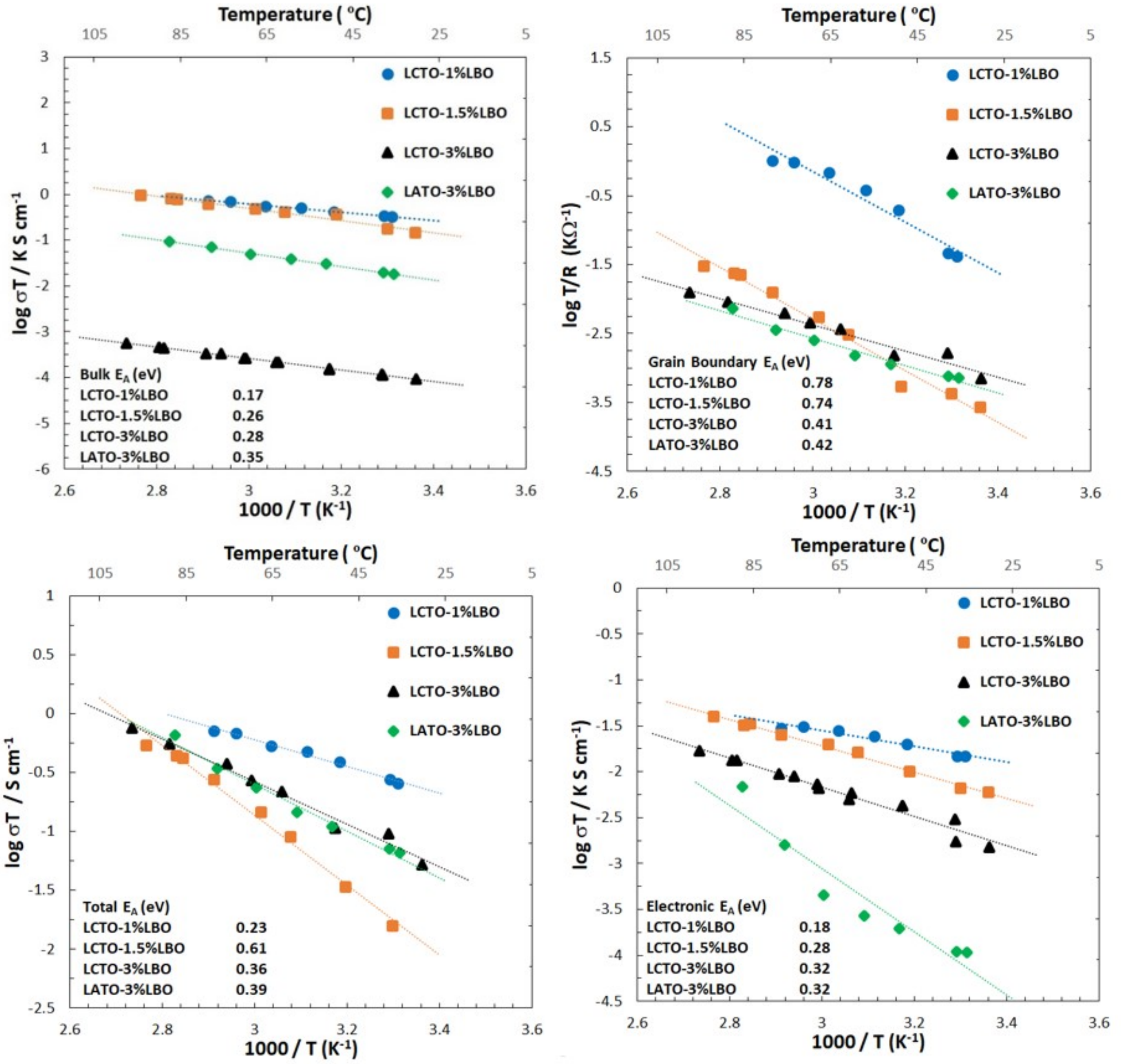

Figure S5. Log (σT) or Log (T/Rgb) plotted as a function of 1/T used for determination of bulk ionic, total ionic, grain boundary and electronic activation energies of $Li_{1.25}CrTi_{1.5}O_4$ (LCTO) and $Li_{1.25}Al_{0.25}Ti_{1.5}O_4$ (LATO) with varied weight percent Li_3BO_3 (LBO) as a function of temperature. E_A is the activation energy, σ is the conductivity, and T is the temperature in Kelvin. Plotting (T/R_{gb}) was done since the grain boundary conductivity cannot be calculated because the grain boundary volume is unknown.

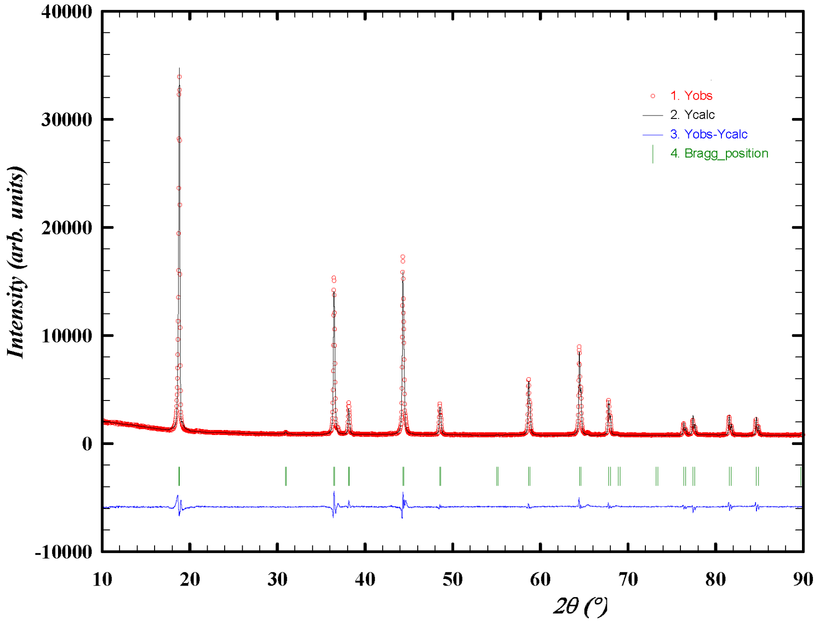

Figure S6. Rietveld fit of $Li_{1.075}Cr_{0.075}Ni_{0.35}Mn_{1.5}O_4$ where red dots are observed points, continuous black line is calculated profile and the positions of Bragg reflections (green) and the observed intensity (Yobs) minus the calculated intensity (Ycalc) difference (blue) are shown at the bottom.

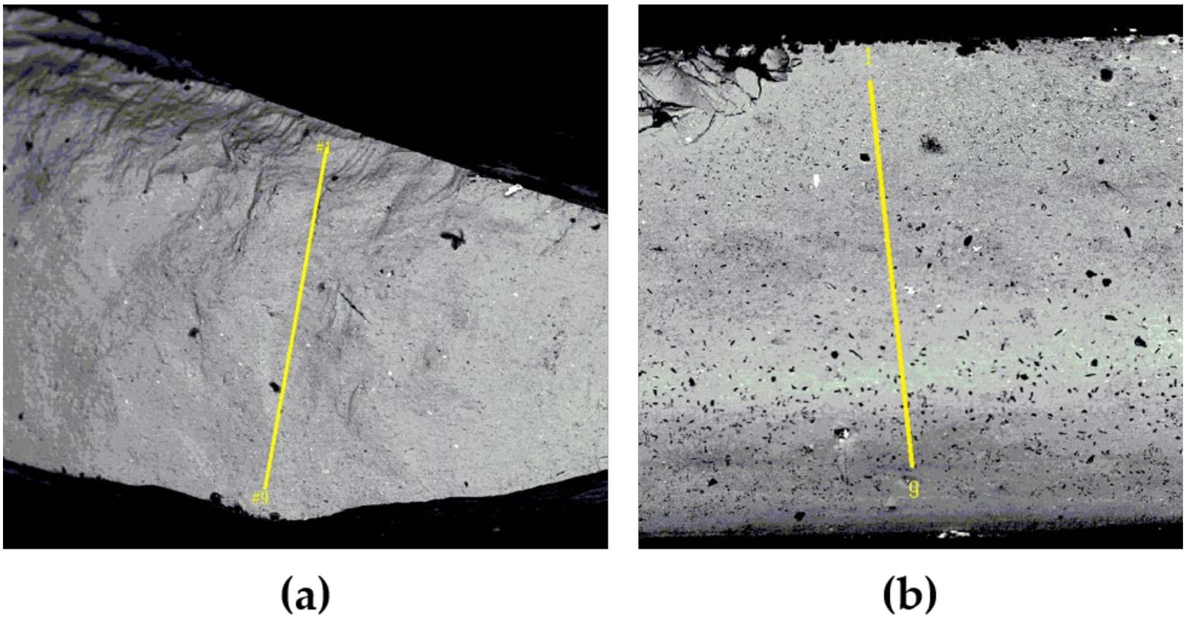

Figure S7. (a) Backscattered Electron Image (BSE) of fractured pellet of $Li_{1.25}Cr_{0.25}Ti_{1.5}O_4$ – 3% Li_3BO_3 used for evaluation of the B distribution. The B_2O_3 equivalent content was measured along nine evenly-spaced spots of 5 μ diameter along a line from spot #1 to spot #9, indicated in yellow on the image. The data is tabulated in Table S2. **(b)** Backscattered Electron Image (BSE) of fractured pellet of $Li_{1.25}Cr_{0.25}Ti_{1.5}O_4$ – 1% Li_3BO_3 used for evaluation of the B distribution. The B_2O_3 equivalent content was measured along nine evenly-spaced spots of 5 μ diameter along a line from spot 1 to spot 9, indicated in yellow on the image. The data is tabulated in Table S2.

Table S1: Final structural model of $Li[Li_{0.25}Cr_{0.25}Ti_{1.5}]O_4$ (LCTO) from Rietveld refinement of X-ray powder diffraction data.

Atom	Wyckoff	x/a	y/a	y/a	B	Occ.
**Li1**	8a	1/8	1/8	1/8	1.81482	1
**Li2**	16d	1/2	1/2	1/2	1.19482	0.25
**Cr**	16d	1/2	1/2	1/2	1.19482	0.25
**Ti**	16d	1/2	1/2	1/2	1.19482	1.5
**O**	32e	0.26321	0.26321	0.26321	1.43259	4

(S.G. F d -3 m); *a* = 8.3440Å

Table S2: Final structural model of $Li[Li_{0.25}Al_{0.25}Ti_{1.5}]O_4$ (LATO)from Rietveld refinement of X-ray powder diffraction data.

Atom	Wyckoff	x/a	y/a	y/a	B	Occ.
**Li1**	8a	1/8	1/8	1/8	1.00106	1
**Li2**	16d	1/2	1/2	1/2	1.00360	0.25
**Al**	16d	1/2	1/2	1/2	1.00360	0.25
**Ti**	16d	1/2	1/2	1/2	1.00360	1.5
**O**	32e	0.26340	0.26340	0.26340	1.74894	4

(S.G. F d -3 m); *a* = 8.3574 Å

Table S3: Lattice constants of $Li_{1.25}Cr_{0.25}Ti_{1.5}O_4$ (LCTO) and $Li_{1.25}Al_{0.25}Ti_{1.5}O_4$ (LATO) pellets as a function of LBO content. Pellets were ground into powders and Si added as internal standard prior to XRD data collection. Lattice constant determined from Rietveld Refinement of the XRD pattern.

Composition	LBO content (wgt. %)	Lattice constant / Å
LCTO	0	8.34444
LCTO	1	8.34559
LCTO	1.5	8.34550
LCTO	3	8.34486
LATO	0	8.34590
LATO	3	8.34739

Table S4: Final parameters of LCTO EIS fit to equivalent circuit.

Circuit element	Value
R1	6805
R2	2.05E+07
CPE2-T	3.5E-10
CPE2-P	0.63
CPE1-T	2.64E-08
CPE1-P	0.62

Table S5: Final structural model of $Li[Li_{0.075}Cr_{0.075}Ni_{0.35}Mn_{1.5}]O_4$ from Rietveld refinement of X-ray powder diffraction data.

Atom	Wyckoff	x/a	y/a	y/a	B	Occ.
**Li1**	8a	1/8	1/8	1/8	0.46242	1
**Li2**	16d	1/2	1/2	1/2	0.49405	0.075
**Cr**	16d	1/2	1/2	1/2	0.49405	0.075
**Ni**	16d	1/2	1/2	1/2	0.49405	0.35
**Mn**	16d	1/2	1/2	1/2	0.49405	1.5
**O**	32e	0.26240	0.26240	0.26240	1.73958	3.87992

(S.G. F d -3 m); a = 8.1704 Å

Table S6. Elemental analysis of LCTO and LATO powders from ICP data compared to nominal stoichiometry of LCTO ($Li_{1.25}Cr_{0.25}Ti_{1.5}O_4$) LATO ($Li_{1.25}Al_{0.25}Ti_{1.5}O_4$).

		**Ratio of total metal ions**			
**Sample**		**Li**	**Cr**	**Al**	**Ti**
LCTO	Nominal	0.417	0.083		0.500
	ICP result	0.417	0.081		0.502
LATO					
	Nominal	0.417		0.083	0.500
	ICP result	0.428		0.073	0.499

Table S7: Boron distribution in $Li_{1.25}Cr_{0.25}Ti_{1.5}O_4$ (LCTO) pellets at two Li_3BO_3 (LBO) contents at positions indicated on **Figures S1 and S2 .**

Composition	Wgt.% LBO	Position	Wgt. % B_2O_3 equivalent
LCTO	3	1	2.54
LCTO	3	2	2.41
LCTO	3	3	2.72
LCTO	3	4	1.26
LCTO	3	5	1.12
LCTO	3	6	2.97
LCTO	3	7	2.17
LCTO	3	8	1.62
LCTO	3	9	1.76
LCTO	3	Average	2.06
LCTO	3	Std. Dev.	0.62
LCTO	1	1	1.31
LCTO	1	2	2.07
LCTO	1	3	2.24
LCTO	1	4	1.72
LCTO	1	5	2.25
LCTO	1	6	2.65
LCTO	1	7	1.31
LCTO	1	8	0.46
LCTO	1	9	0.43
LCTO	1	Average	1.60
LTO	1	Std. Dev.	0.75
